# Dendrosomal Curcumin Showed Cytotoxic Effects on Breast Cancer Cell Line by Inducing Mitochondrial Apoptosis Pathway and Cell Division Arrest

**DOI:** 10.5812/ijpr-151714

**Published:** 2024-10-13

**Authors:** Houriye Abbasi, Fatemeh Hosseinkhani, Bahareh Imani Fouladi, Siroos Tarighi, Majid Sadeghizadeh, Maryam Montazeri

**Affiliations:** 1 Department of Biotechnology, Faculty of Advanced Sciences and Technology, Tehran Medical Sciences, Islamic Azad University, Tehran, Iran; 2 Department of Cellular and Molecular, Faculty of Advanced Sciences and Technology, Tehran Medical Sciences, Islamic Azad University, Tehran, Iran; 3 Department of Genetics, Faculty of Bio Sciences, Tarbiat Modares University, Tehran, Iran

**Keywords:** Cancer, Nanocurcumin, Apoptosis, Gene, Protein

## Abstract

**Background:**

Mutations in the *p53 gene* have been linked to the initiation and progression of breast cancer, as well as resistance to chemotherapy. Therefore, the development of novel treatment approaches is essential to combat this disease.

**Objectives:**

This study aimed to evaluate the effects of dendrosomal curcumin (DNC) on the breast cancer cell line MDA-MB231.

**Methods:**

MDA-MB231 cells were treated with 20 μM DNC, and the apoptosis rate and cell proliferation cycles were assessed using flow cytometry. Additionally, after RNA extraction and cDNA synthesis, the expression levels of *Lnc-DANCR*, *EZH2*, *Noxa*, *bcl-2*, *bax*, *PUMA*, *p21*, and *p53 gene*s were analyzed using RT-PCR. Protein expression levels of *P53*, *P21*, *Bcl-2*, and *Bax* were evaluated through western blotting.

**Results:**

Dendrosomal curcumin induced apoptosis in MDA-MB231 cells and caused cell cycle arrest at the SubG1 phase. Dendrosomal curcumin treatment downregulated *Lnc-DANCR*, *EZH2*, *bcl-2*, and *p53 gene* expression, while upregulating *bax*, *Noxa*, *PUMA*, and *p21* gene expression in a time-dependent manner. *Bax* and *P21* protein levels were significantly upregulated following DNC treatment, whereas *Bcl-2* and *P53* protein levels were downregulated in DNC-treated breast cancer cells.

**Conclusions:**

In summary, dendrosomal nanocurcumin demonstrated potent anti-tumor effects against breast cancer cells, suggesting its potential as a therapeutic agent in breast cancer treatment.

## 1. Background

Breast cancer is diagnosed in approximately a quarter of all women with cancer and causes the death of around 570,000 patients annually (1). Early diagnosis is crucial in reducing mortality and improving patient survival (2). One of the primary risk factors for this malignancy is genetic mutations, particularly in the apoptosis pathway, which allows cancer cells to resist apoptosis and contribute to tumor development (3). The polyproline region of the *P53 gene* plays a vital role in tumor suppressor activity (4), promoting apoptosis by releasing various pro-apoptotic factors (5). Consequently, mutations in this domain are linked to the development of multiple cancers (6). Notably, *P53 gene* mutations are more frequently observed in breast cancer, with over 80% of triple-negative breast cancer (TNBC) cases exhibiting such mutations (7). The MDA-MB-231 cell line (a TNBC cell model) has a *P53 gene* mutation known as *R280K* (8). It is important to note that TNBC patients are often resistant to chemotherapy and have a lower survival rate (9). Therefore, the development of new treatment strategies for this disease is essential.

Curcumin is a natural compound known for its wide range of pharmacological properties, including antioxidant (10), analgesic (11), anti-inflammatory (12), antimicrobial (13), anti-mutagenic (14), and anti-tumor effects (15). It has demonstrated anticancer activity against various malignancies, including head and neck squamous cell carcinoma (HNSCC) (16), prostate (17), colorectal (18), breast (19), and brain cancers (20). Mechanisms underlying curcumin's anticancer effects include downregulation of NF-κB (21), induction of apoptosis through increased oxidative stress, upregulation of *P53* (22), and inhibition of CXCL1/2 production (23). However, its therapeutic use has been hindered by poor intestinal absorption, bioavailability, and water solubility (24).

Recent advancements in nanotechnology have addressed these limitations by developing curcumin-loaded nanoformulations. For example, Montazeri et al. (2016) introduced dendrosomal curcumin (DNC), which possesses amphiphilic properties and high bioavailability, and demonstrated its anticancer effects on hepatocellular carcinoma by inducing apoptosis and inhibiting cell proliferation (24). Dendrosomal curcumin's effects on breast cancer have also been investigated. For instance, DNC was found to inhibit metastasis in breast cancer by modulating immune responses, upregulating *STAT4* and *IL-2*, and reducing the expression of STAT3 and *IL-10* genes (25). Moreover, DNC induced apoptosis in MCF7 cells (26), and downregulation of Lnc*HOTAIR* was proposed as one of its mechanisms of action (27). Additionally, a combination of DNC with exogenous *P53* in the MDA-MB-231 cell line induced cell death via apoptosis and decreased the expression levels of *ZEB1* and *BMI1* genes (28).

Despite these promising findings, further studies are needed to fully elucidate DNC's mechanisms of action in breast cancer. 

## 2. Objectives

This study aimed to investigate the effects of DNC on the MDA-MB-231 cell line and evaluate changes in gene and protein expression related to cell cycle regulation and mitochondrial apoptosis processes.

## 3. Methods

### 3.1. Dendrosomal Curcumin and Cell Preparations 

Dendrosomal curcumin was generously provided by Babaei et al., who previously synthesized and evaluated DNC's effects on the mouse fibrosarcoma cell line WEHI-164 (29). MDA-MB231 cells were cultured immediately in DMEM medium containing 1% penicillin/streptomycin and 10% FBS (GIBCO, USA) and incubated in a humidified atmosphere of 5% CO_2_ at 37°C. The cells (80 × 10⁶ cells/mL) were seeded into 12-well plates and treated with 20 µM DNC (30) and free curcumin for 240 minutes. To assess curcumin uptake by the cells, fluorescence microscopy was used (24)

### 3.2. Cell Viability 

MDA-MB231 cells (4 × 10⁴ cells/mL) were seeded into a 96-well plate with 200 µL of DMEM medium and incubated overnight under the conditions described above. The following day, the cells were treated with 20 µM DNC and incubated for three days. Afterward, 5 mg/mL of MTT solution was added, and the cells were incubated for 240 minutes. Finally, the upper layer was removed, and 200 µL of DMSO was added. After 15 minutes, the optical density (OD) was measured using an ELISA reader at 490 nm.

### 3.3. Cell Proliferation Cycles 

The effects of DNC on MDA-MB231 cell proliferation cycles were evaluated using the propidium iodide (PI) staining method and flow cytometry. Briefly, 3 × 10⁵ cells/mL were exposed to 20 µM DNC for different time periods. The cells were washed with PBS and fixed with 75% ethanol for 15 minutes at 4°C, then washed again with PBS. The cells were treated with PI (50 µg/mL in PBS) for 15 minutes and analyzed using a FACSCalibur flow cytometer (Becton Dickinson, USA).

### 3.4. Apoptosis 

The percentage of apoptotic cells in DNC-treated samples was measured using flow cytometry and Annexin V staining, following the manufacturer's instructions with the Annexin-V-FITC kit (Roche, Germany).

### 3.5. Gene Expression 

We used an RNA extraction kit (Invitrogen, USA) for isolating total RNA based on the instructions provided by the manufacturer. After ensuring the high quality and quantity of extracted RNA, cDNA was synthesized using a commercial kit (Takara, Japan). The primers specific for Lnc-DANCR (F-5'-CCTCAGTTCTTAGCGCAGGTTG-3, R-5'-ACTGCTCTAGCTCCTGTGGC-3'); EZH2 (F-5'- CACGGGGATAGAGAATGTGGGTT -3', R-5'- AGTTCTTCTGCTGTGCCCTTATCTG -3); bax (F-5'- GTGGATGACTGAGTACCTGAAC -3', R-5'- GCCAGGAGAAATCAAACAGAGG -3); Noxa (F-5'- GAGCTGGAAGTCGAGTGTG -3', R-5'- CTCTTTTGAAGGAGTCCCCTC -3); PUMA (F-5'- GAGATGGAGCCCAATTAGGTG -3', R-5'- ACATGGTGCAGAGAAAGTCC -3); p21 (F-5'- CCTGTCACTGTCTTGTACCC -3', R-5'- GTGGTAGAAATCTGTCATGCTG -3); p53 (F-5'- TCCTCAGCATCTTATCCGAGTG -3', R-5'- AGGACAGGCACAAACACGCACC -3); bcl-2 (F-5'- GAGCAGATCATGAAGACAGGG -3', R-5'- ATGCGCTTGAGACACTCG -3) and GAPDH (R-5'- GTGAACCATGAGAAGTATGACAAC-3', R-5'- CATGAGTCCTTCCACGATACC-3') were designed using Oligo software and blasted on the NCBI website. The reaction mixture for RT-PCR included 0.5 µL of each F and R primer, 5 µL Master Mix, 0.75 ng cDNA, and 3.5 µL deionized water. The time-temperature program of the RT-PCR device (Applied Biosystems, USA) was 1 cycle of 95°C for 15 min, 40 cycles of 95°C for 5 s, 63°C for 23 s, and 72°C for 34 s, with a final cycle of 72°C for 10 min. The 2^-ΔΔCT^ method was used for gene expression data analysis.

### 3.6. Western Blot 

Proteins from DNC-treated cells were extracted using RIPA buffer via the immunoprecipitation method and quantified by the Bradford assay. Extracted proteins were separated by SDS-PAGE and transferred to nitrocellulose membranes. The membranes were blocked with 5% skim milk for 90 minutes and incubated overnight at 4°C with primary antibodies for β-actin, *Bcl-2, P21, P53*, and *bax* proteins. The membranes were then washed with TBST and incubated with secondary goat HRP anti-mouse IgG. The chemiluminescence method and ECL kit were used to visualize the protein bands.

### 3.7. Statistical Analysis 

Data were analyzed using the ANOVA procedure, and Tukey's post hoc test was used for mean comparisons at P < 0.05. The analyses were conducted using GraphPad Prism V.8 software.

## 4. Results

### 4.1. Uptake Kinetics 

**Figure 1.**
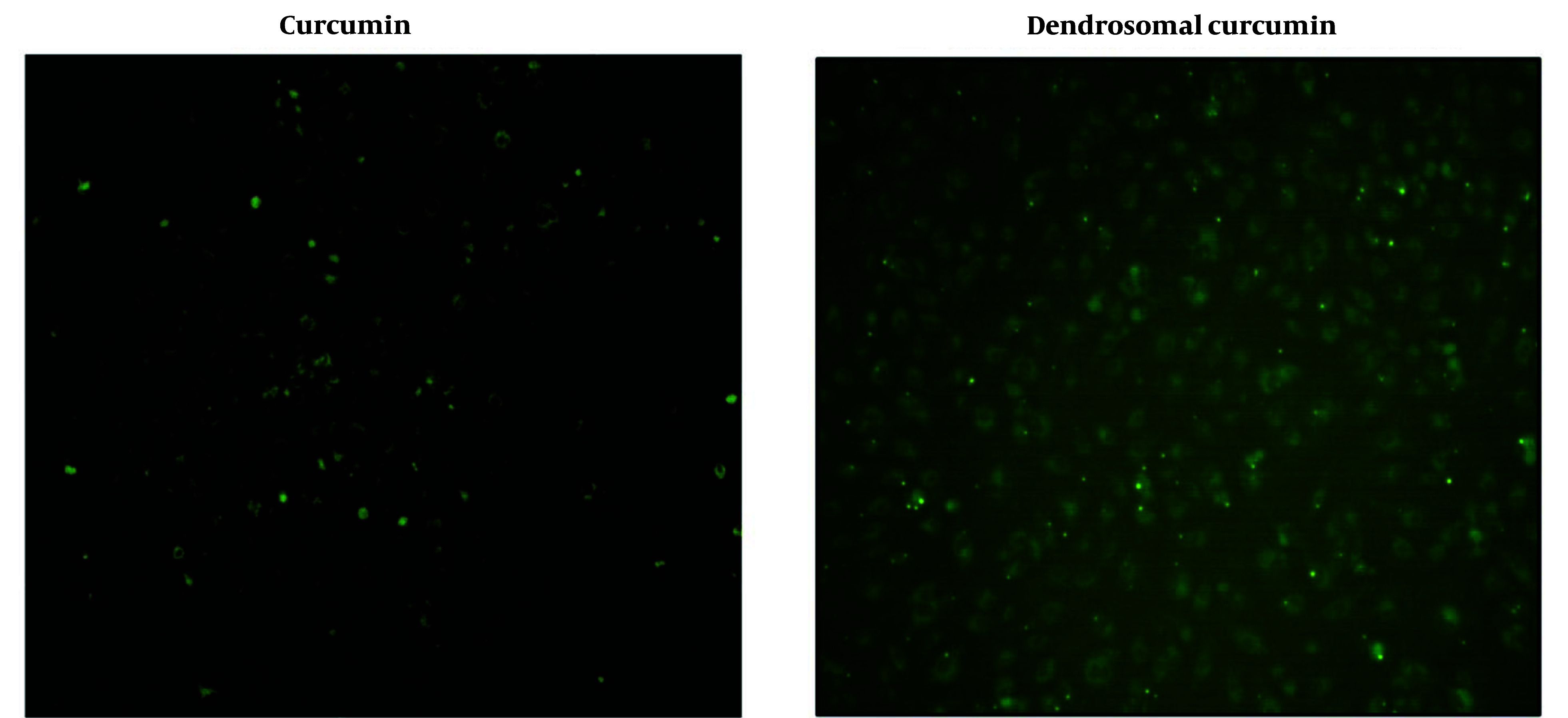
The uptake of curcumin and dendrosomal curcumin (DNC) by MDA-MB-231 cell line. The cells were treated with 20 µM DNC for 240 min and seen under fluorescence microscopy.

The uptake of dendrosomal curcumin compared to bulk curcumin was assessed using fluorescence microscopy, leveraging curcumin's inherent fluorescent properties. In this experiment, cells were cultured in 24-well plates, and after 24 hours, they were treated with 20 μM curcumin and 20 μM DNC. Four hours post-treatment, the cells were examined under a fluorescence microscope. The results demonstrated that DNC exhibited significantly higher penetration efficiency into the cells compared to bulk curcumin (Figure 1). 

### 4.2. Cell Viability, Apoptosis, and Necrosis 

**Figure 2.**
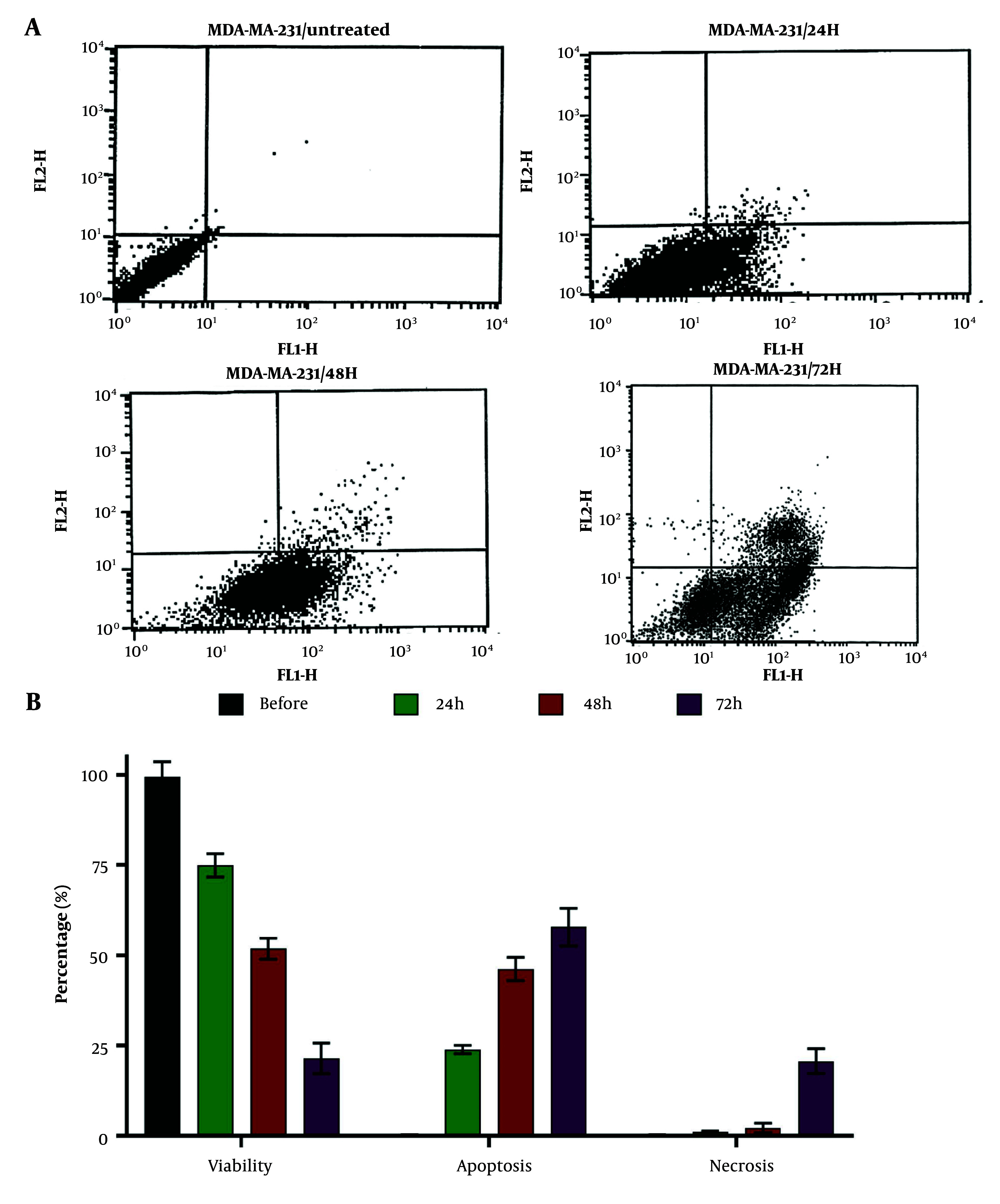
A, the histograms obtained by flowcytometry; B, the impacts of dendrosomal curcumin (DNC, 20 μM) on MDA-MB-231 cells' viability, apoptosis and necrosis before treatment and 24, 48 and 72 hours after (n = 3).

Dendrosomal curcumin (20 μM) reduced the cell viability of MDA-MB231 breast cancer cells in a time-dependent manner. Before treatment, cell viability was measured at 99.51 ± 4.11%, and 72 hours after DNC treatment, it decreased to 21.45 ± 4.22% (Figure 2), highlighting the potent anticancer effects of this nanoformulation. The primary cause of cell death was apoptosis, with 57.80 ± 5.22% of cells undergoing apoptosis 72 hours post-treatment. In comparison, the percentage of necrotic cells was 20.7 ± 3.44% at the same time point (Figure 2), indicating that DNC predominantly induces cell death through apoptosis.

### 4.3. Cell Cycles 

**Figure 3.**
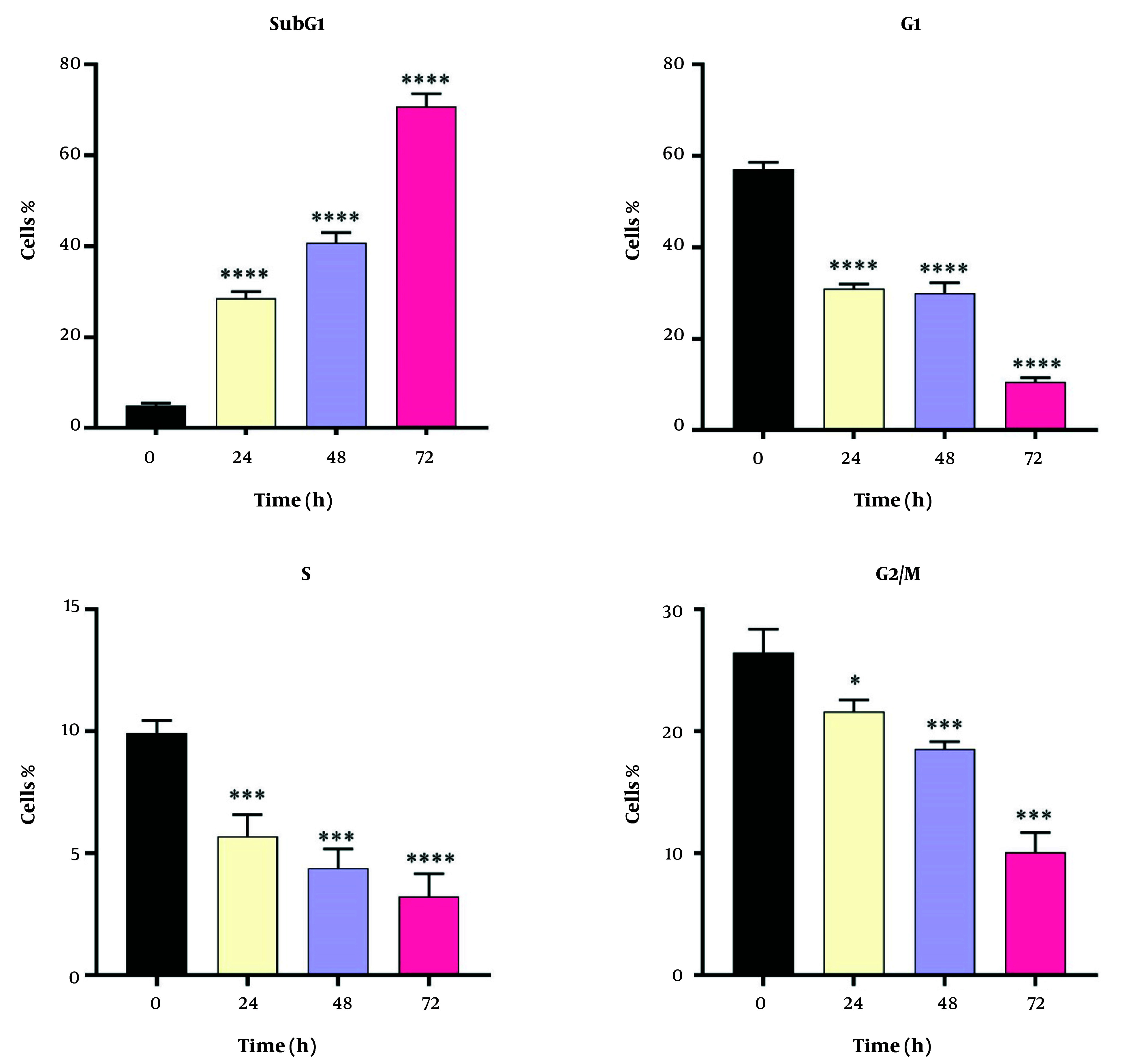
The effects of dendrosomal curcumin (DNC, 20 μM) on the MDA-MB-231 cells proliferation cycle at SubG1, G1, S and G2/M stages (n = 3); * P < 0.05; *** P < 0.001; **** P < 0.0001.

**Figure 4.**
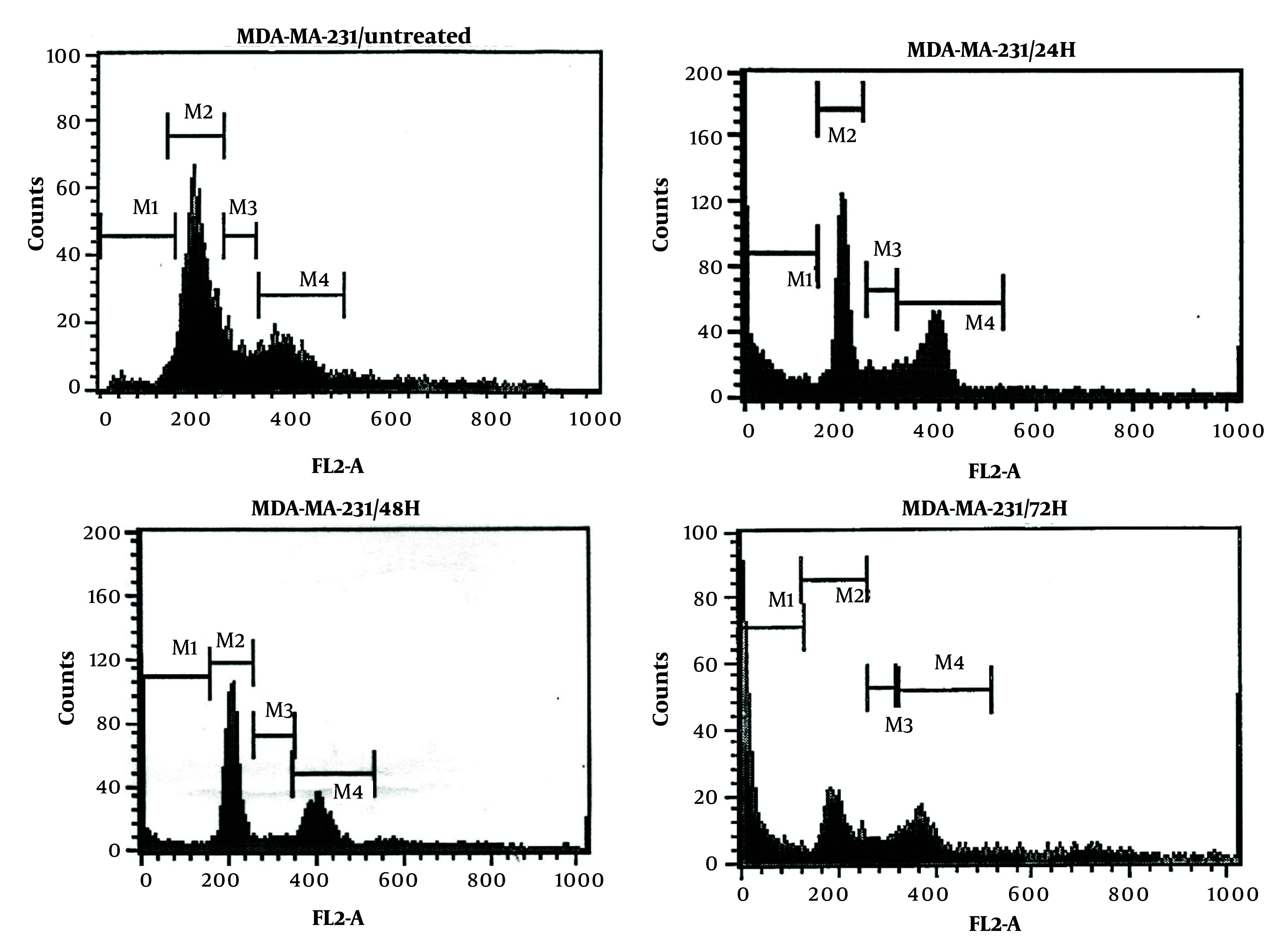
The histograms obtained by flow-cytometry for evaluating the impacts of dendrosomal curcumin (DNC, 20 μM) during 0, 24, 48 and 72 h on the MDA-MB-231 cells proliferation cycle at SubG1, G1, S and G2/M stages (n = 3).

Dendrosomal curcumin treatment arrested MDA-MB231 cancer cells at the SubG1 phase of the cell cycle, effectively preventing cell division. As depicted in Figure 3, approximately 70% of DNC-treated cells were in the SubG1 phase 72 hours post-treatment. The inhibition of cell division began 24 hours after DNC exposure and persisted for the entire 72-hour period. These results further confirm the anti-tumor effects of DNC against MDA-MB231 cells. The flow cytometry histograms illustrating these findings are shown in Figure 4. 

### 4.4. Gene Expression Analysis 

**Figure 5.**
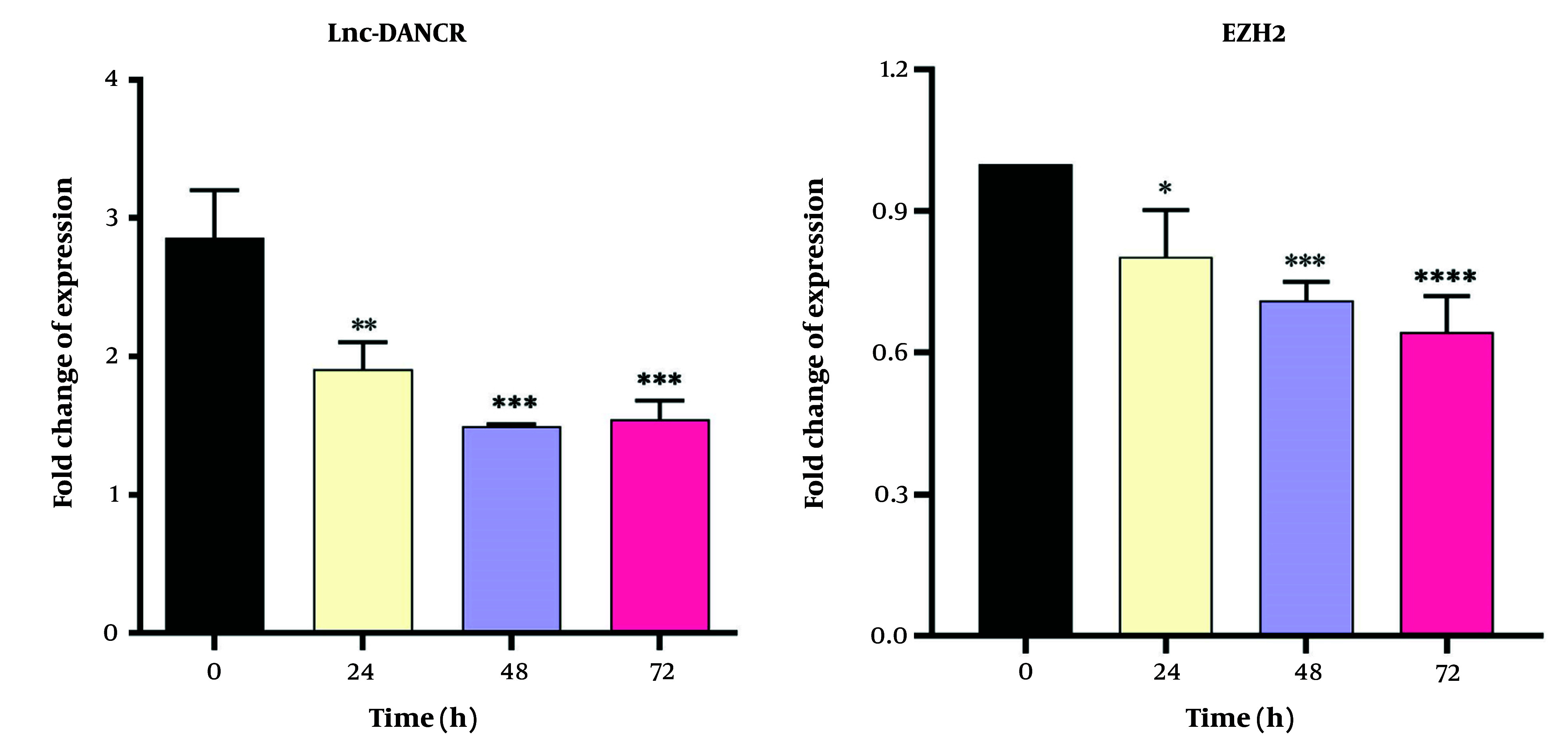
The impacts of dendrosomal curcumin (DNC, 20 μM) on expressions of *Lnc-DANCR* and *EZH2* genes of MDA-MB-231 cells (n = 3). *GAPDH* gene was used as the housekeeping gene. **** P < 0.0001; *** P < 0.001; ** P < 0.01; * P < 0.05.

Dendrosomal curcumin treatment downregulated the expression of both *Lnc-DANCR* and *EZH2* genes in a time-dependent manner in MDA-MB231 cells. The lowest expression levels of *Lnc-DANCR* were observed 48 and 72 hours after DNC treatment (Figure 5). Similarly, the lowest expression levels of the *EZH2* gene were also recorded at these time points (Figure 5). These findings suggest that DNC effectively suppresses the expression of key genes involved in tumor progression.

**Figure 6.**
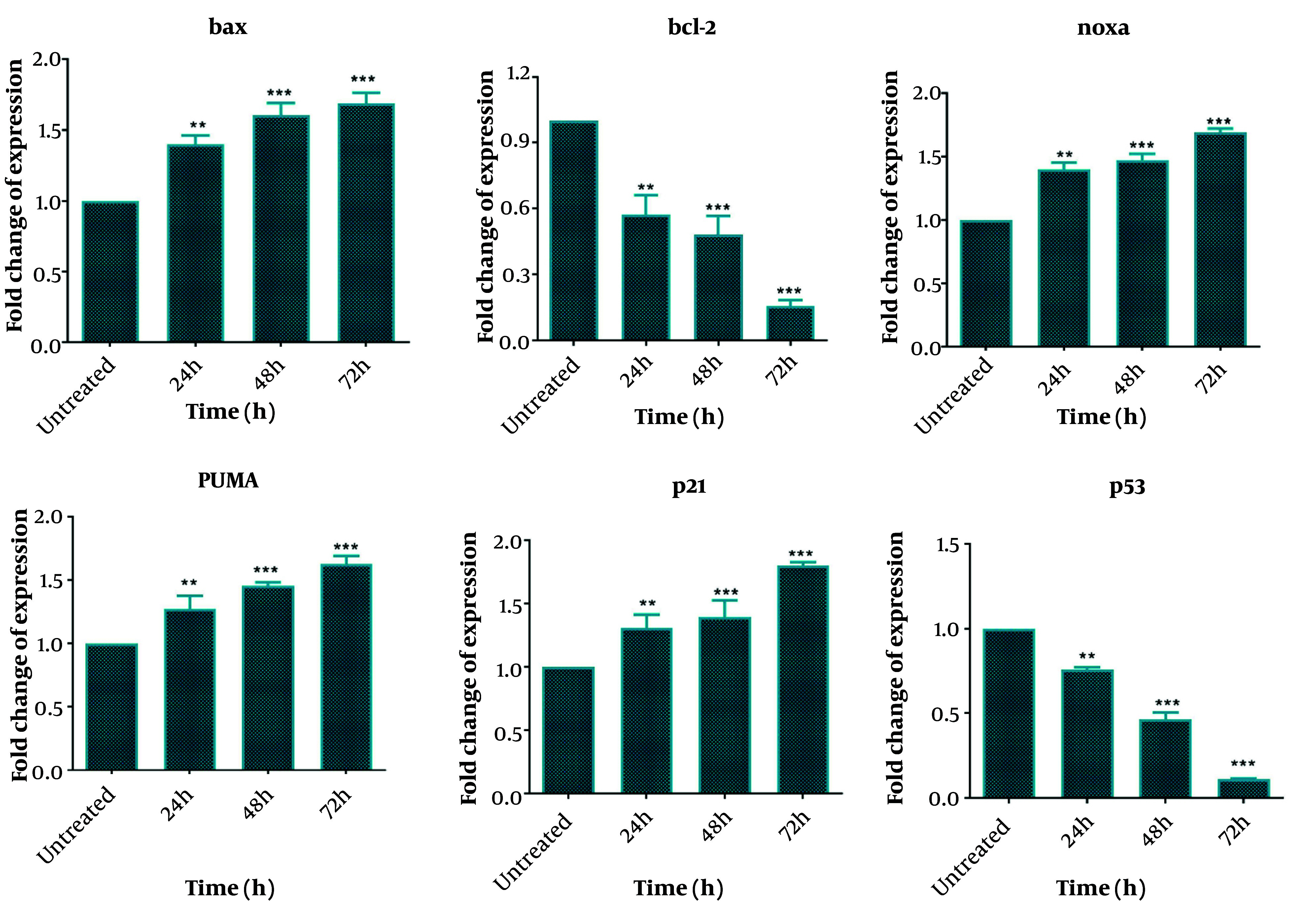
The impacts of dendrosomal curcumin (DNC, 20 μM) on expressions of *bcl-2*, *bax*, *Noxa*, *PUMA*, *p21* and *p53 gene*s of MDA-MB-231 cells (n = 3). *GAPDH* gene was used as the housekeeping gene. **** P < 0.0001; *** P < 0.001; ** P < 0.01.

One day after DNC treatment, significant changes were observed in the expression levels of *bcl-2*, *bax*, *Noxa*, *PUMA*, *p21*, and *p53 gene*s, with these changes becoming more pronounced over time (up to 72 hours). Specifically, the expressions of *bax*, *Noxa*, *PUMA*, and *p21* genes increased significantly in a time-dependent manner following DNC treatment compared to untreated cells. In contrast, the expressions of *bcl-2* and *p53 gene*s were significantly downregulated compared to untreated cells (Figure 6). These results highlight the role of DNC in regulating key genes involved in apoptosis and cell cycle arrest.

### 4.5. Western Blot Analyses 

**Figure 7.**
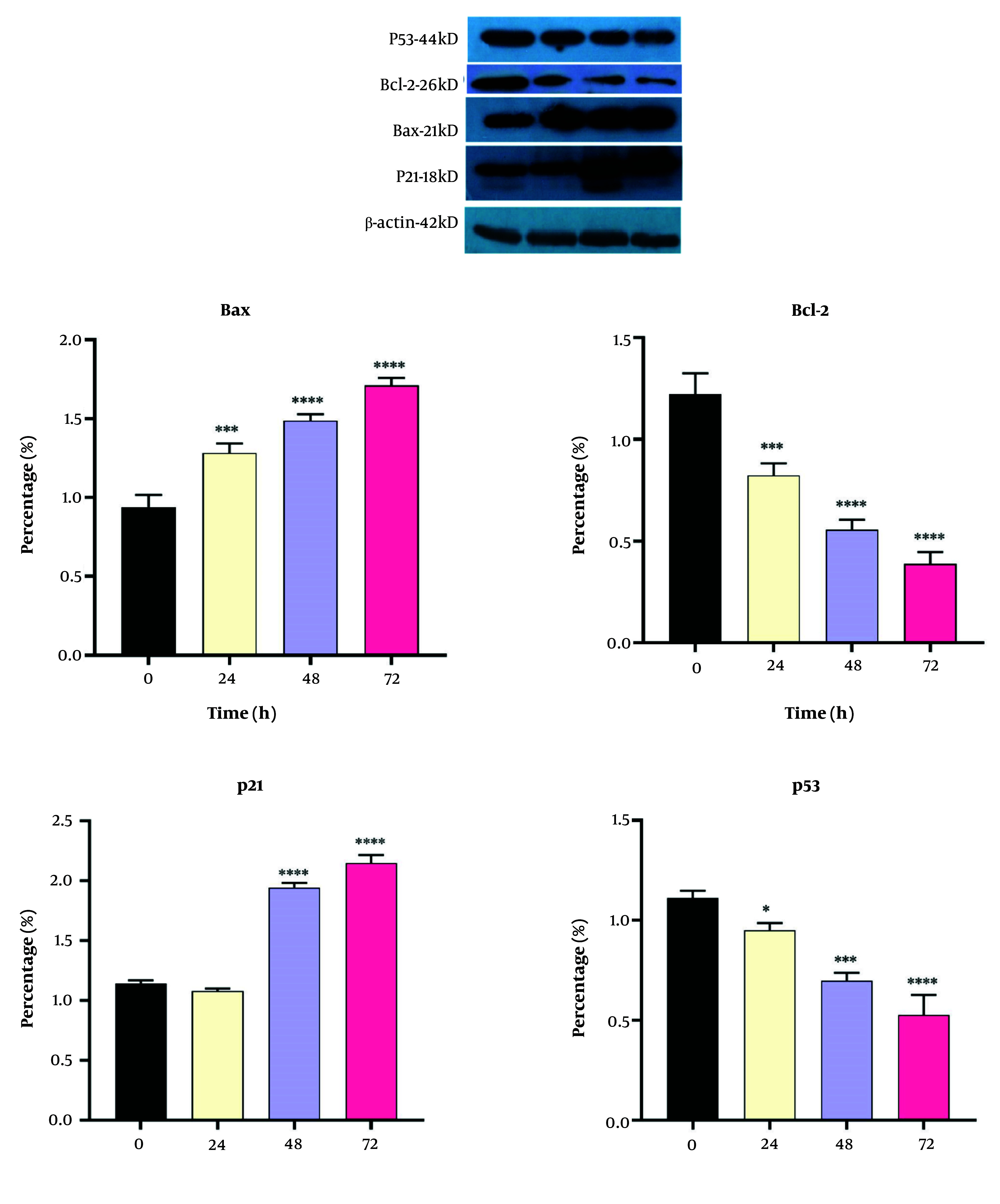
The expressions of *Bcl-2, Bax, P53 and P21* proteins in the MDA-MB-231 cells before and after treatment with dendrosomal curcumin (DNC, 20 μM) (n = 3). **** P < 0.0001; *** P < 0.001; * P < 0.05.

We also measured the expressions of Bcl-2, Bax, P21, and P53 proteins in the DNC-treated cells, and the results are shown in Figure 7. As can be seen, Bax and P21 were significantly upregulated after DNC treatment; however, both Bcl-2 and P53 were downregulated in the 20 µM DNC-exposed MDA-MB-231 cells.

## 5. Discussion

The findings of this study indicated improved penetration of curcumin when loaded into dendrosomes, significantly enhancing its entry into MDA-MB-231 breast cancer cells and inducing apoptosis. Additionally, DNC effectively arrested the cells in the SubG1 phase of mitotic division, preventing cell proliferation. Dendrosomal curcumin treatment also led to decreased expression of *Lnc-DANCR* and *EZH2* genes, while upregulating *bax*, *Noxa*, *PUMA*, and *p21* genes and downregulating *bcl-2* and *p53* in the breast cancer cell line. Western blot analysis further confirmed downregulation of Bcl-2 and P53 proteins and upregulation of Bax and P21 in DNC-treated cells.

Curcumin is a natural compound with extensive pharmacological properties, including anticancer effects (31). However, its low solubility and poor intestinal absorption have been significant barriers to fully utilizing its therapeutic potential (32). One solution to this issue has been the development of curcumin-loaded nanoformulations (32), which enhance curcumin’s solubility through amphipathic properties. In this study, DNC uptake was evaluated in breast cancer cells, and the results demonstrated that DNC showed superior cell entry compared to free curcumin, likely due to improved solubility in its dendrosomal form (29).

Furthermore, DNC significantly reduced the viability of breast cancer cells, demonstrating its strong potential to induce apoptosis. Similar findings were reported by Baghi et al. (2018), who highlighted DNC’s anticancer effects in breast cancer cells through apoptosis induction, with cytotoxicity being enhanced by exogenous *p53* (28). In another study, DNC demonstrated cytotoxic effects on T47D and MCF-7 breast cancer cells by inducing apoptosis (33). Seyed Hosseini et al. (2023) also reported DNC’s anticancer effects against ovarian cancer, upregulating matrix metalloproteinase-2 (MMP-2) (34). These researchers further observed decreased expression of *HOTAIR*, *bcl-2*, and *H19*, along with overexpression of *MEG3*, after treating ovarian cancer cells with DNC (35), indicating that curcumin exerts its anticancer effects via multiple pathways.

In summary, DNC shows great potential as an adjuvant therapy in cancer treatment by inducing apoptosis in cancerous cells.

Dendrosomal curcumin -treated cells exhibited cell division arrest at the SubG1 phase, suggesting that curcumin disrupts the mitotic process. This finding aligns with other studies reporting the arrest of cancer cells, such as Huh/HepG2, in the SubG1 and G2/M phases after curcumin treatment (24, 36), demonstrating curcumin's ability to inhibit cancer cell development by disrupting mitosis. Additionally, G2/M phase arrest has been observed in pancreatic (37) and head and neck squamous carcinoma cell lines (38). The ATM/Chk2-*P53* axis is thought to play a key role in this process (38), while curcumin-induced cell cycle arrest also involves inhibition of NF-κB and histone deacetylase 4 (39, 40).

*Lnc-DANCR* is known to be upregulated in advanced breast cancer and is associated with metastasis (41). The MDA-MB-231 cell line, used in this study, is highly malignant and metastatic. Here, we observed high expression of the *Lnc-DANCR* gene in these cells, which significantly decreased 48 hours after DNC treatment. Previous research has shown that *Lnc-DANCR* knockdown leads to breast cancer cell cycle arrest in the G0/G1 phase (41). Thus, the arrest of DNC-treated cells in the SubG1 phase may be linked to the reduction in *Lnc-DANCR* expression. *Lnc-DANCR* knockdown prevents *EZH2* from binding to the SOCS3 promoter, leading to SOCS3 upregulation, which in turn inhibits tumor growth (41, 42). Therefore, the downregulation of *Lnc-DANCR* and *EZH2* in DNC-treated cells further explains the nanoformulation's anticancer effects.

We also evaluated the gene and protein expressions related to the intrinsic apoptotic pathway, including *bax*, *Noxa*, *PUMA*, *p21*, *bcl-2*, and *p53*. Cancer cells often evade programmed cell death through the attenuation of the apoptosis pathway, making the induction of apoptosis a key mechanism in anticancer therapies (37). Many chemotherapy drugs exert their effects by inducing apoptosis (43). In this study, DNC effectively induced apoptosis in breast cancer cells, showcasing its potential as an antitumor agent. The gene expression analysis revealed that DNC upregulated *bax*, *Noxa*, *PUMA*, and *p21*, while downregulating *bcl-2* and *p53*. Correspondingly, at the protein level, DNC increased *Bax and P21 expressions and decreased Bcl-2 and P53* levels. *P53* plays a central role in both intrinsic and extrinsic apoptosis by stimulating the production of pro-apoptotic proteins such as *Bax*, *Noxa*, and *PUMA*, which promote cytochrome C release and lead to cell death (44). Furthermore, the reduction of *Bcl-2* expression is associated with increased apoptosis. Thus, the upregulation of pro-apoptotic genes and proteins, and the downregulation of anti-apoptotic factors, indicates the activation of the intrinsic apoptosis pathway in DNC-treated cells. Notably, the *P53* protein in the MDA-MB-231 cell line is a mutant form with potential oncogenic activity (45), and the observed reduction in both mRNA and protein levels of *P53* after DNC treatment may explain the anticancer effects seen in this study.

### 5.1. Conclusions

In conclusion, dendrosomal nanocurcumin exhibited strong anti-tumor effects against breast cancer cells. The mechanisms of action were linked to cell division arrest at the SubG1 phase and the induction of apoptosis by upregulating pro-apoptotic factors, including *bax, Noxa, and PUMA, while downregulating the anti-apoptotic factor bcl-2. Fu*rther investigation of the anticancer effects of DNC in animal models is recommended to validate its potential as a therapeutic agent for cancer treatment.

## Data Availability

The dataset presented in the study is available on request from the corresponding author during submission or after publication.
